# Methylphenidate Enhances NMDA-Receptor Response in Medial Prefrontal Cortex via Sigma-1 Receptor: A Novel Mechanism for Methylphenidate Action

**DOI:** 10.1371/journal.pone.0051910

**Published:** 2012-12-20

**Authors:** Chun-Lei Zhang, Ze-Jun Feng, Yue Liu, Xiao-Hua Ji, Ji-Yun Peng, Xue-Han Zhang, Xue-Chu Zhen, Bao-Ming Li

**Affiliations:** 1 Institute of Neurobiology and State Key laboratory of Medical Neurobiology, Institutes of Brain Science, Fudan University, Shanghai, China; 2 Center for Neuropsychiatric Disorders, Institute of Life Science, Nanchang University, Nanchang, China; 3 Neuropharmacological Laboratory, State Key Laboratory of Drug Research, Shanghai Institute of Materia Medica, Chinese Academy of Sciences, Shanghai, China; University of Medicine & Dentistry of NJ - New Jersey Medical School, United States of America

## Abstract

Methylphenidate (MPH), commercially called Ritalin or Concerta, has been widely used as a drug for Attention Deficit Hyperactivity Disorder (ADHD). Noteworthily, growing numbers of young people using prescribed MPH improperly for pleasurable enhancement, take high risk of addiction. Thus, understanding the mechanism underlying high level of MPH action in the brain becomes an important goal nowadays. As a blocker of catecholamine transporters, its therapeutic effect is explained as being due to proper modulation of D1 and α2A receptor. Here we showed that higher dose of MPH facilitates NMDA-receptor mediated synaptic transmission via a catecholamine-independent mechanism, in layer V∼VI pyramidal cells of the rat medial prefrontal cortex (PFC). To indicate its postsynaptic action, we next found that MPH facilitates NMDA-induced current and such facilitation could be blocked by σ1 but not D1/5 and α2 receptor antagonists. And this MPH eliciting enhancement of NMDA-receptor activity involves PLC, PKC and IP3 receptor mediated intracellular Ca^2+^ increase, but does not require PKA and extracellular Ca^2+^ influx. Our additional pharmacological studies confirmed that higher dose of MPH increases locomotor activity via interacting with σ1 receptor. Together, the present study demonstrates for the first time that MPH facilitates NMDA-receptor mediated synaptic transmission via σ1 receptor, and such facilitation requires PLC/IP3/PKC signaling pathway. This novel mechanism possibly explains the underlying mechanism for MPH induced addictive potential and other psychiatric side effects.

## Introduction

Methylphenidate (MPH, known as Ritalin or Concerta), is a commonly used stimulant medication for Attention-deficit/hyperactivity disorder (ADHD) [1], [2]. As acutely administered MPH has a good safety profile, and improves executive function performance in both diagnosed ADHD patients and general healthy population [3]–[6], its prescription has been strikingly increased nowadays. However, these young people using prescribed MPH improperly for pleasurable enhancement, have high risk of being addicted [7].

In the ADHD patients, the symptoms are mostly consistent with the dysfunction of the PFC [8], [9], where is a high-function area guiding and organizing attention, thought and affection [10]. As a blocker of dopamine (DA) and norepinephrine (NE) transporters [11], [12], low to moderate levels of MPH increase both extracellular DA and NE in PFC [13], and DA in the striatum [14]. Interestingly, a recent animal study showed that low dose of MPH infusion into PFC facilitates working memory performance, while MPH into striatum does not affect this PFC-dependent cognition task [15]. Thus, these evidence support the notion that PFC is a main site involving in MPH’s therapeutic actions [1], [16].

Through strengthening DA/NE transmission in PFC, low to moderate doses of MPH have been shown to improve working memory in animals [13], [17], [18]. Importantly, recent electrophysiological studies explored more on the receptor mechanisms for MPH actions. For example, *in vivo* acutely administered MPH exerts excitatory actions on PFC neurons by indirectly activating α2-adrenoceptors and D1 receptors [1], [17]–[19]. And *in vitro*, MPH could enhance excitability of pyramidal PFC neurons by activating α2 receptors located in interneurons [20].

On the other hand, escalating or higher doses of MPH induce behavioral sensitization, like locomotor hyperactivity in animals [21], [22], which is associated with a robust DA release in the PFC, caudate-putamen, and nucleus accumbens, etc [1], [14], [23]. Among these brain areas, a series of studies by Dafny et al indicated that PFC is still an important area for acute or chronic administration of MPH induced sensitization in free-moving animals [24]–[26]. Moreover, higher doses or long-term medication of MPH may lead some psychiatric adverse effects like depressive symptoms both in animals and patients [27]–[29]. Although higher doses of MPH actions may be due to excessive stimulation on D1, α1 and/or β1 receptors [1], its explicit receptor mechanism underlying the side effects like addiction and other psychiatric effects remains mostly unexplored.

Interestingly, recent studies have indicated that sigma-1 (σ1) receptor is a new target for those stimulants used for ADHD, like cocaine [30], [31], amphetamine [32]–[34], and 3,4-methylenedioxymethamphetamine (MDMA) [35]. σ receptor was first described as a subtype of opioid receptor [36] or phencyclidine (PCP) receptor [37]. As σ receptor is not the high-affinity binding site of naltrexone or thienylcyclohexylpiperidine (TCP), it was re-classified as a non-opioid receptor [38] or non-PCP receptor [39], [40]. Two subtypes of σ receptor have been described: σ1 and σ2 receptors [41]. By measuring the mRNA level in the brain, sigma-1 receptor protein is highly distributed in the PFC, striatum and hippocampus, etc [42]. On the cellular level, σ1 receptor showing a post-synaptic distribution, is enriched in the endoplasmic reticulum and on the plasma membrane through the dynamic translocation [42]. Activation of the σ1 receptor would modulate Ca^2+^ entry through plasma membrane (i.e., via K^+^ channel, NMDA receptor, voltage-sensitive Ca^2+^ channel), and intracellular Ca^2+^ mobilization (i.e., via IP3 receptor) [43], [44].

Considering MPH’s important role in regulating PFC function and ADHD medication, our study attempted to characterize the pharmacological and cellular mechanism of MPH in layer V∼VI pyramidal cells in the medial prefrontal cortex of rats. We found that MPH facilitates NMDA-receptor mediated excitatory synaptic transmission through σ1 receptors via PLC/PKC signaling pathway, revealing a novel mechanism for MPH action.

## Results

Whole-cell patch clamp recordings were conducted in layer V∼VI pyramidal cells in slices of rat mPFC. Pyramidal cells were identified by their morphological and electrophysiological features. These neurons have pyramidal-shaped cell bodies and long apical dendrites extending toward superficial layers, as revealed by IR-DIC. They had a resting membrane potential more negative than −60 mV and an action potential larger than 70 mV, with no spontaneous discharge. They exhibited a spike frequency adaptation in response to a depolarizing current pulse and could be characterized as “regular spiking” pyramidal cells [45].

### MPH Enhances NMDA- and Non-NMDA-R Mediated Synaptic Transmission

**Figure 1:**
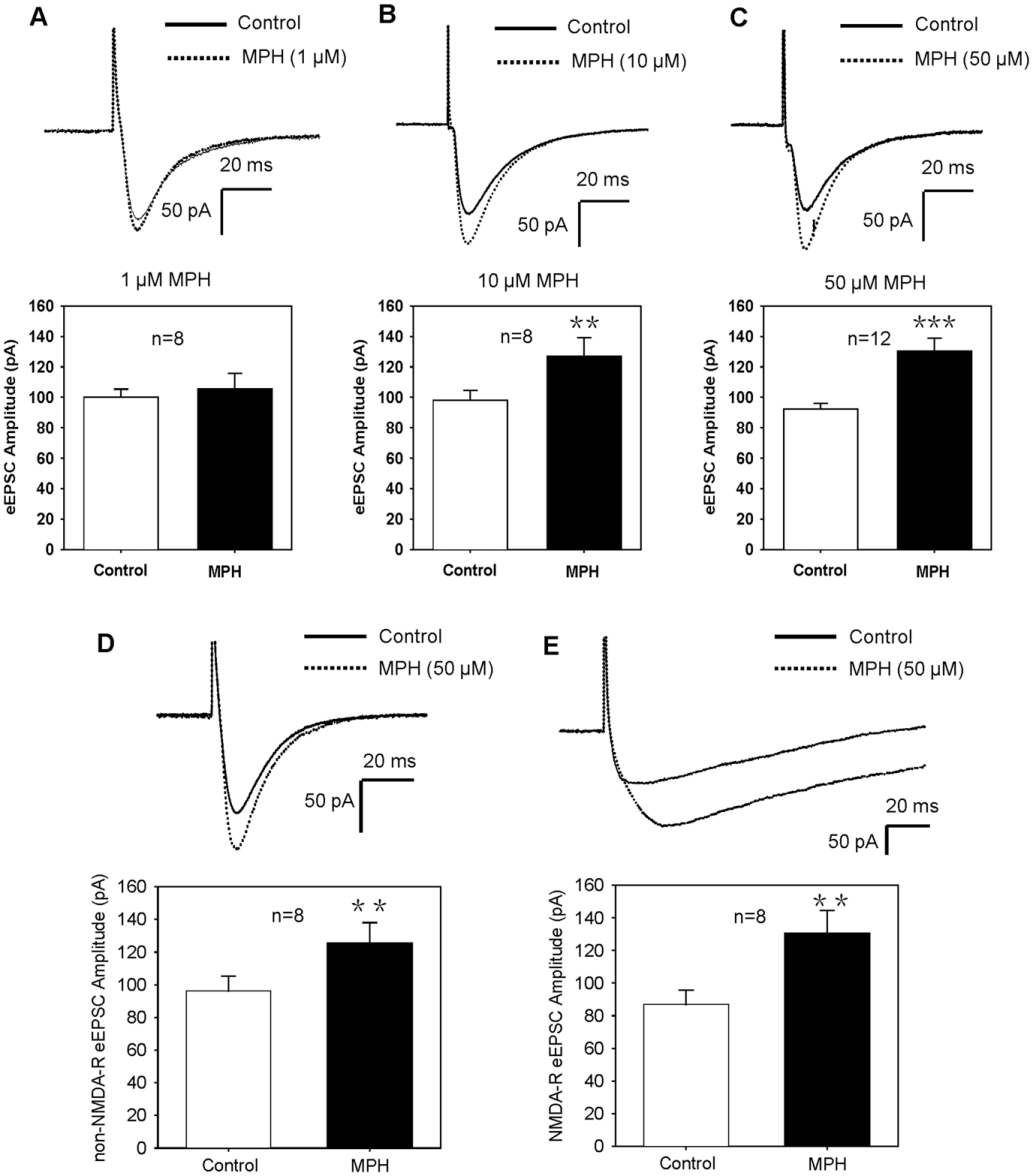
MPH enhances both non-NMDA- and NMDA-R mediated eEPSC. (**A**) MPH with 1 µM had no effect on the amplitude of eEPSC. P>0.05 for MPH vs. control, n = 8, paired t-test. (**B**) MPH with 10 µM significantly enhanced the amplitude of eEPSC. **P<0.01 vs. control, n = 8, paired t-test. (**C**) MPH with 50 µM significantly enhanced the amplitude of eEPSC. ***P<0.001 vs. control, n = 12, paired t-test. (**D**) MPH (50 µM) enhanced non-NMDA-R mediated eEPSC. Recordings of eEPSC were carried out in the presence of AP-5 (50 µM; NMDA receptor antagonist), with holding potential of −70 mV. **P<0.01 vs. control, n = 8, paired t-test. (**E**) MPH (50 µM) enhanced NMDA-R mediated eEPSC. Recordings of eEPSCs were performed in the presence of CNQX (20 µM; non-NMDA receptor antagonist), with holding potential of −40 mV to relieve the voltage-dependent Mg^2+^ blockade of NMDA receptor. **P<0.01 vs. control, n = 8, paired t-test. All traces of the synaptic currents are the average of 10 consecutive eEPSC responses. Recordings of eEPSCs were conducted in the continuous presence of BMI, with holding potential of −70 mV (A–D) or −40 mV (E).

We first tested if treatment with methylphenidate hydrochloride (MPH) could affect excitatory synaptic transmission in pyramidal cells. Recordings of eEPSC were conducted in the continuous presence of the GABAergic antagonist bicuculline (BMI). Under voltage-clamp at a holding potential of −70 mV, eEPSCs were evoked at a stimulation rate of 0.033 Hz. These eEPSCs could be completely inhibited by co-application of AP-5 (50 µM) and CNQX (20 µM) (data not shown), indicating that the currents were mediated by ionotropic glutamate receptors. As shown in Figure 1B and 1C, bath application of MPH (10, 50 µM) significantly enhanced eEPSC. While MPH with 1 µM produced no effect (Figure 1A, 105.2±8.9% of the baseline eEPSC, n = 8, paired t-test, P>0.05), MPH with 10 µM significantly enhanced eEPSC (129.5±8.6% of the baseline eEPSC, n = 8, P<0.01), and such enhancement was more evident when MPH dose was 50 µM (142.0±9.3% of the baseline eEPSC, n = 12, P<0.001).

To characterize if MPH enhancement came from a facilitation of NMDA-receptor (NMDA-R) or non-NMDA-receptor (non-NMDA-R) components, or both, we examined the effects of MPH on NMDA-R and non-NMDA-R mediated eEPSC, respectively. When non-NMDA-R current was recorded, we held the membrane potential at −70 mV and applied AP-5 (50 µM) to block NMDA-R. The non-NMDA-R eEPSC could be blocked wholly by the non-NMDA-R antagonist CNQX (20 µM) (data not shown). When NMDA-R current was recorded, we held the membrane potential at −40 mV (to relieve the voltage-dependent Mg^2+^ blockade of NMDA-R channel) and applied CNQX (20 µM) to block non-NMDA-R. NMDA-R mediated eEPSC could be blocked completely by the NMDA-R antagonist AP-5 (50 µM) (data not shown). As shown in Figure 1D and 1E, MPH (50 µM) significantly enhanced both non-NMDA- and NMDA-R mediated eEPSC (non-NMDA-R eEPSC: 131.0±5.2% of baseline, n = 8, P<0.01; NMDA-R eEPSC: 151.6±11.9% of baseline, n = 8, P<0.01, paired t-test).

### MPH Facilitates Excitatory Synaptic Transmission via Pre- and Postsynaptic Mechanisms

As a blocker of dopamine (DA) and norepinephrine (NE) transporters, MPH could increase the concentrations of DA and NE in synaptic cleft [14]. Thus, MPH enhancement of eEPSC may be a result of enhanced synaptic transmission mediated by catecholamine. If so, the facilitation effect of MPH should not exist after catecholamine is depleted.

**Figure 2:**
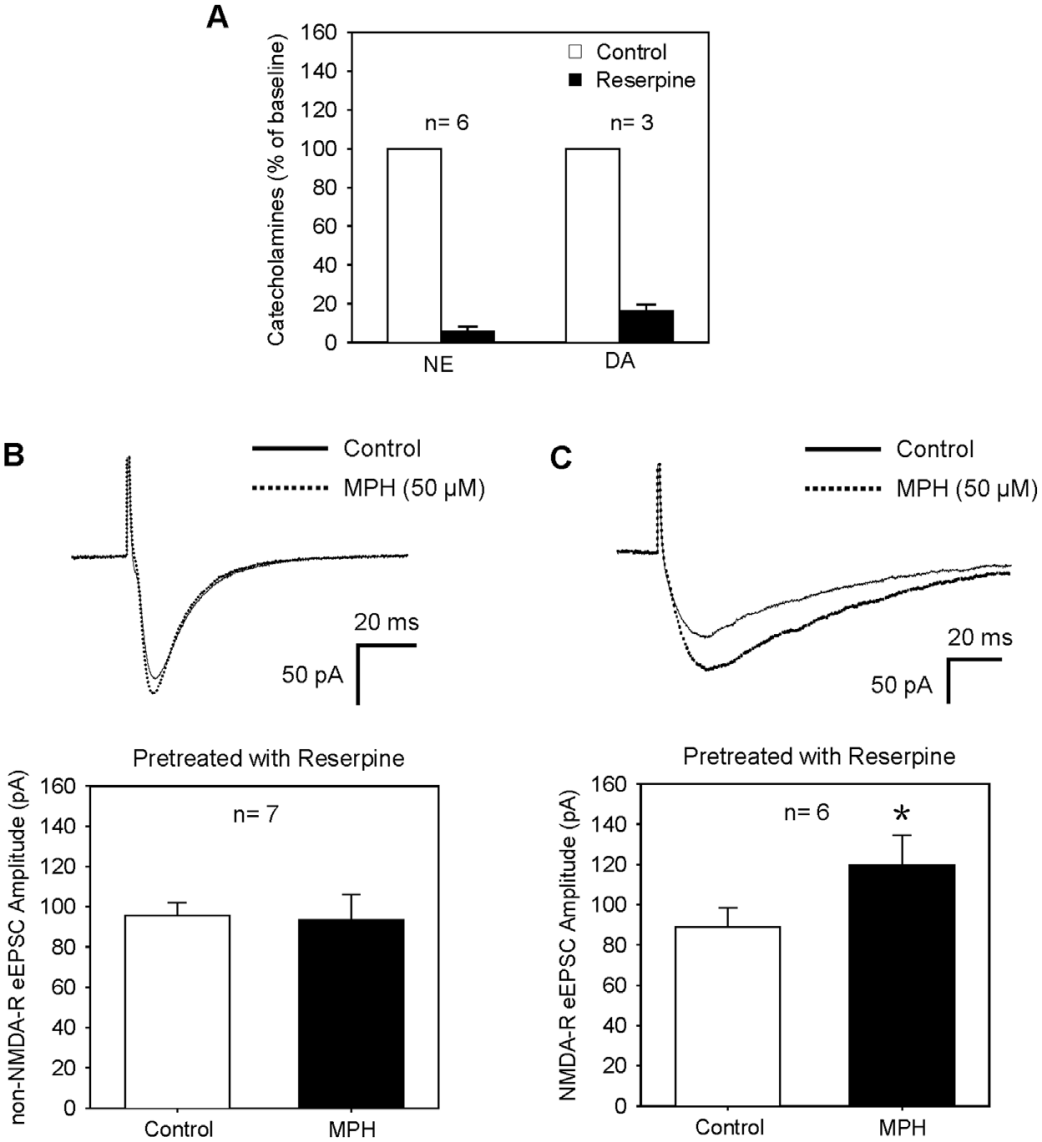
MPH enhances NMDA-R mediated eEPSC under depletion of catecholamine. (**A**) The levels of NE and DA were almost completely depleted in slices treated with reserpine. n = 6 slices for NE, and n = 3 slices for DA. (**B**) In reserpine-treated slices, MPH (50 µM) produced no effect on non-NMDA-R mediated eEPSC. Recordings of eEPSCs were performed in the presence of AP-5 (50 µM), with holding potential of −70 mV. P>0.05 for MPH vs. control, n = 7, paired t-test. (**C**) In reserpine-treated slices, MPH (50 µM) still enhanced NMDA-R mediated eEPSC. Recording of eEPSCs were performed in the presence of CNQX (20 µM), with holding potential of −40 mV. *P<0.05 vs. control, n = 6, paired t-test.

In this experiment, we used reserpine, an inhibitor of the vesicular monoamine transporter, to deplete catecholamine (see the methods). As shown in Figure 2A, NE and DA were almost completely depleted in reserpine-treated slices (6.0±2.1% of baseline for NE, n = 6 slices; and 16.1±3.4% for DA, n = 3 slices). In such slices, MPH produced no effect on non-NMDA-R eEPSC (97.1±9.8% of the baseline eEPSC, n = 7, P>0.05) (Figure 2B), but still enhanced NMDA-R eEPSC (135.5±16.2% of the baseline eEPSC, n = 6, P<0.05) (Figure 2C). Thus, MPH enhances non-NMDA-R eEPSC via a catecholamine-dependent mechanism (presynaptic mechanism), whereas it facilitates NMDA-R eEPSC through a catecholamine-independent way.

**Figure 3:**
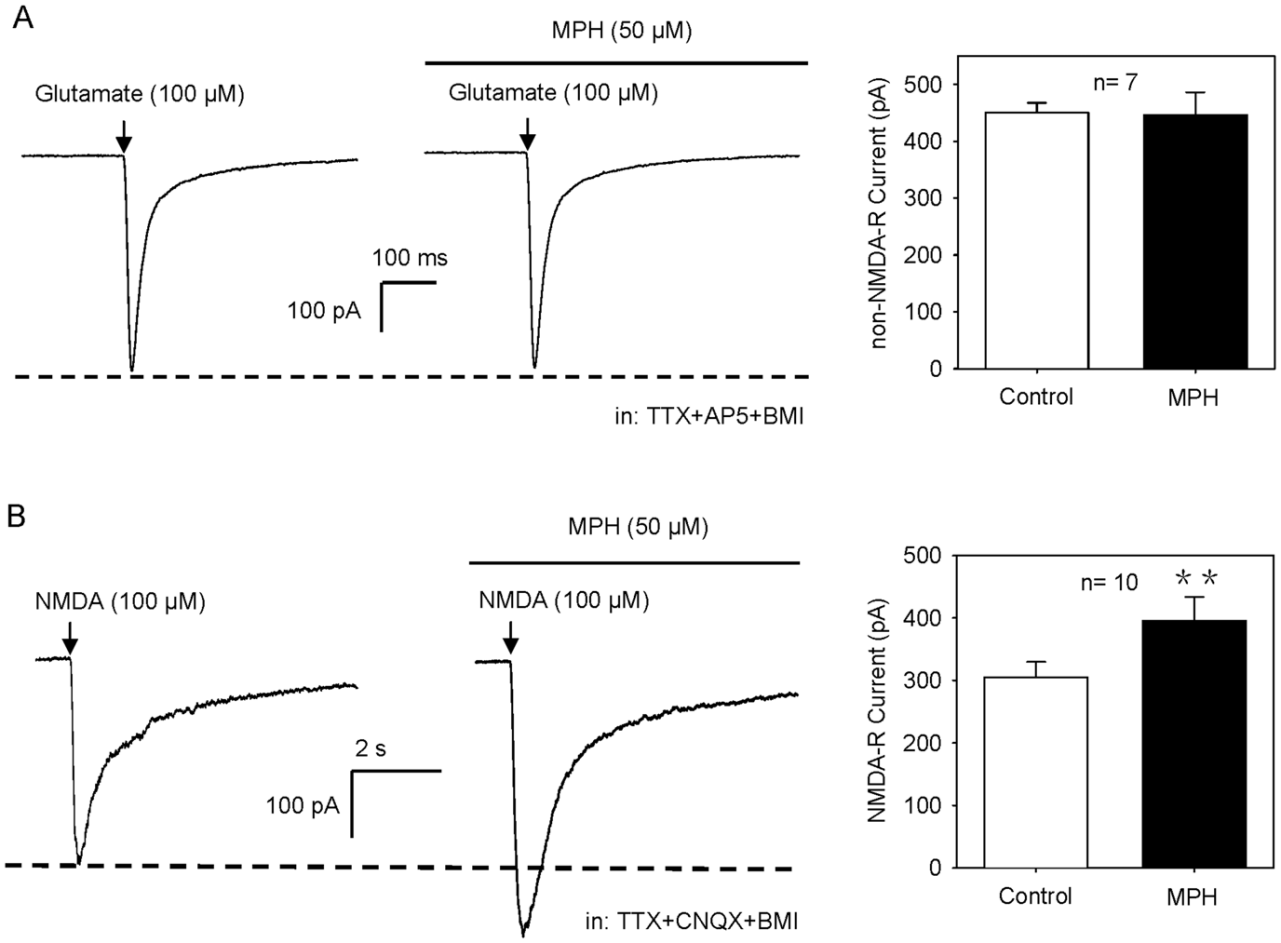
MPH has no effect on non-NMDA-R current, but enhances NMDA-R current in pharmacologically-isolated cells. (**A**) MPH (50 µM) produced no effect on non-NMDA-R current. Recordings of non-NMDA-R currents were performed in the presence of AP-5 (50 µM), TTX (1 µM) and BMI (20 µM), with holding potential of −70 mV. As seen, pressure-application of glutamate (100 µM) induced an inward non-NMDA-R current (*left*), and this current was unchanged when MPH was administered (*right*). P>0.05 for MPH vs. control, n = 7, paired t-test. (**B**) MPH (50 µM) enhanced NMDA-R current. Recordings of NMDA-R currents were performed in the presence of CNQX (20 µM), TTX (1 µM) and BMI (20 µM), with holding potential of −40 mV. As shown, pressure-application of NMDA (100 µM) induced an inward NMDA-R current (*left*), and this current was enhanced when MPH was applied (*right*). **P<0.01 vs. control, n = 10, paired t-test.

To future confirm this notion, we pharmacologically isolated the patched cells by bath applying TTX and BMI, and puff administered glutamate to induce non-NMDA-R current or NMDA to induce NMDA-R current. The non-NMDA- and NMDA-R currents could be eliminated by CNQX and AP-5, respectively. As shown in Figure 3A, MPH had no effect on non-NMDA-R current (98.4±6.5% of the baseline, n = 7, P>0.05, paired t-test), but significantly enhanced NMDA-R current (129.6±6.2% of the baseline, n = 10, P<0.01) (Figure 3B), indicating that there exists a post-synaptic mechanism mediating MPH facilitation of NMDA-R current.

### MPH Enhances NMDA-R Response through σ1 but not D1/5 and α2 Receptors

**Figure 4:**
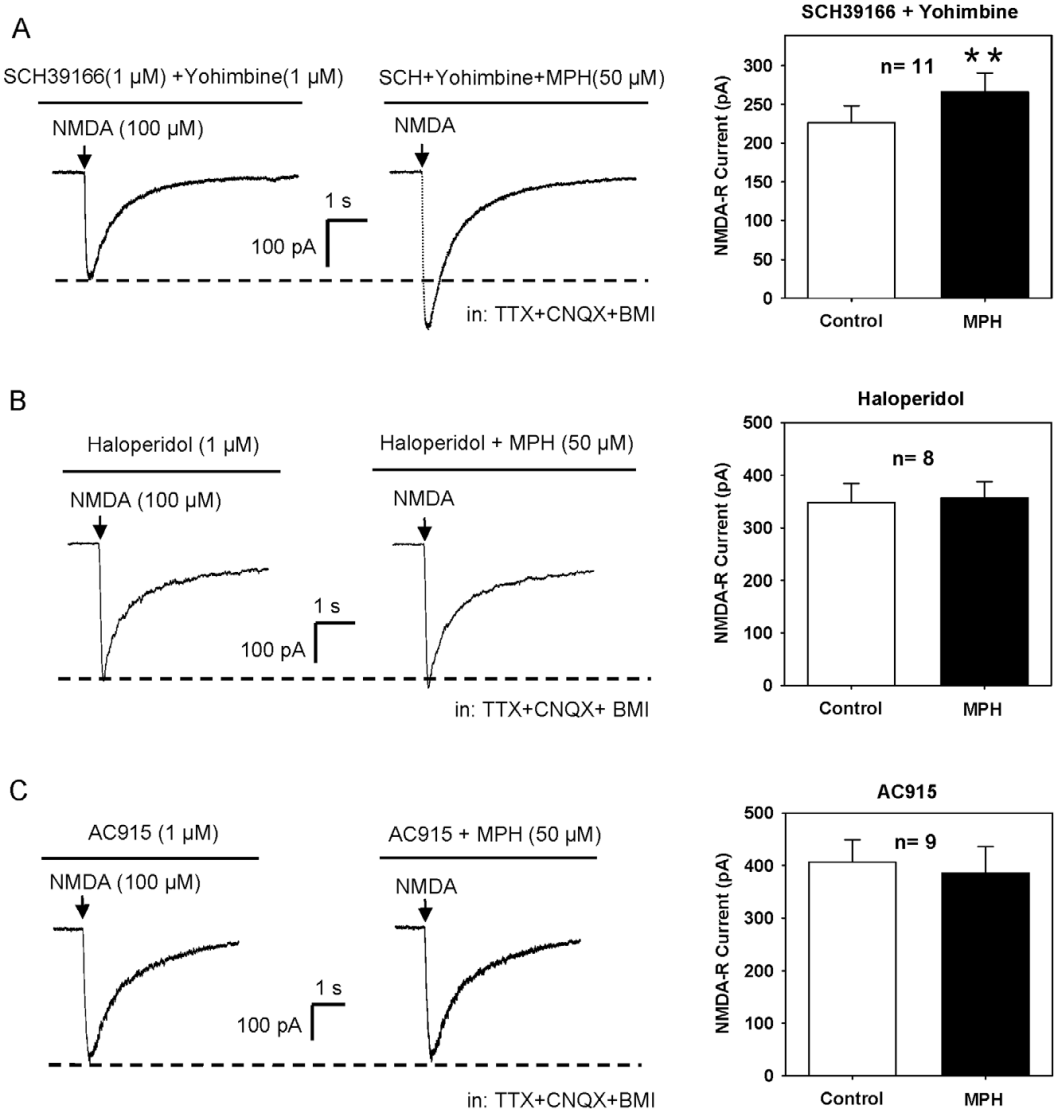
MPH enhancement of NMDA-R current is mediated by σ1, but not D1/5 and α2 receptors. (**A**) MPH (50 µM) still enhanced NMDA-induced current when the D1/5 receptor antagonist SCH39166 (1 µM) and the α2 receptor antagonist yohimbine (1 µM) were co-administered. **P<0.01 vs. control, n = 11, paired t-test. (**B**) MPH (50 µM) did not enhance NMDA-induced current in the presence of the potent σ1 receptor antagonist haloperidol (1 µM). P>0.05 for MPH vs. control, n = 8, paired t-test. (**C**) MPH (50 µM) did not enhance NMDA-induced current in the presence of the selective σ1 receptor antagonist AC915 (1 µM). P>0.05 for MPH vs. control, n = 9, paired t-test. NMDA-R currents were recorded in the presence of CNQX (20 µM), TTX (1 µM) and BMI (20 µM), with holding potential of −40 mV.

It is important to know the receptor mechanism underlying MPH facilitation of NMDA-R mediated synaptic transmission. Behavioral pharmacological studies have shown that MPH improves prefrontal cortical cognitive functions through actions at NE α2 and DA D1 receptors [1], [17]–[19]. It has been reported that MPH increases the excitability of PFC pyramidal neurons via activation of α2 receptors [20]. Moreover, stimulation on D1 receptors has been shown to potentiate NMDA-R current in rat PFC [46]. Thus, we tested if D1 and/or α2 receptors involve in MPH enhancement of NMDA-R current. As shown in Figure 4, MPH still enhanced NMDA-R current when the D1/5 antagonist SCH39166 and the α2 antagonist yohimbine were co-administered (119.4±6.0% of the baseline, n = 11, P<0.01) (Figure 4A) or applied separately (data not shown). Thus, MPH enhancement of NMDA-R response was not directly mediated by D1 and α2 receptors.

Previous studies have shown that stimulation of σ1 receptor regulates NMDA-R mediated intracellular calcium elevation, NMDA-R current and NMDA-R mediated synaptic transmission [44], [47], [48]. Thus, we speculated that MPH enhancement of NMDA-R current might have something to do with σ1 receptor. To address this speculation, we investigated MPH effect in the presence of haloperidol (1 µM), a potent σ1 receptor antagonist [49], and found that MPH facilitation of NMDA-R current did not appear when haloperidol was bath applied (104.0±5.3% of the baseline, n = 8, P>0.05) (Figure 4B).

Since haloperidol is also a D2 receptor antagonist, we then examined the effect of AC915, a selective σ1 receptor antagonist, to further confirm the role of σ1 receptor. As shown in Figure 4C, MPH enhancement of NMDA-R response was blocked in the presence of AC915 (1 µM) (93.7±3.4% of the baseline, n = 9, P>0.05). Taken together, these results indicate that MPH could act at σ1 receptor to enhance NMDA-R response.

### Competitive Binding Assays Reveal that MPH could Bind with σ1 Receptor

**Figure 5:**
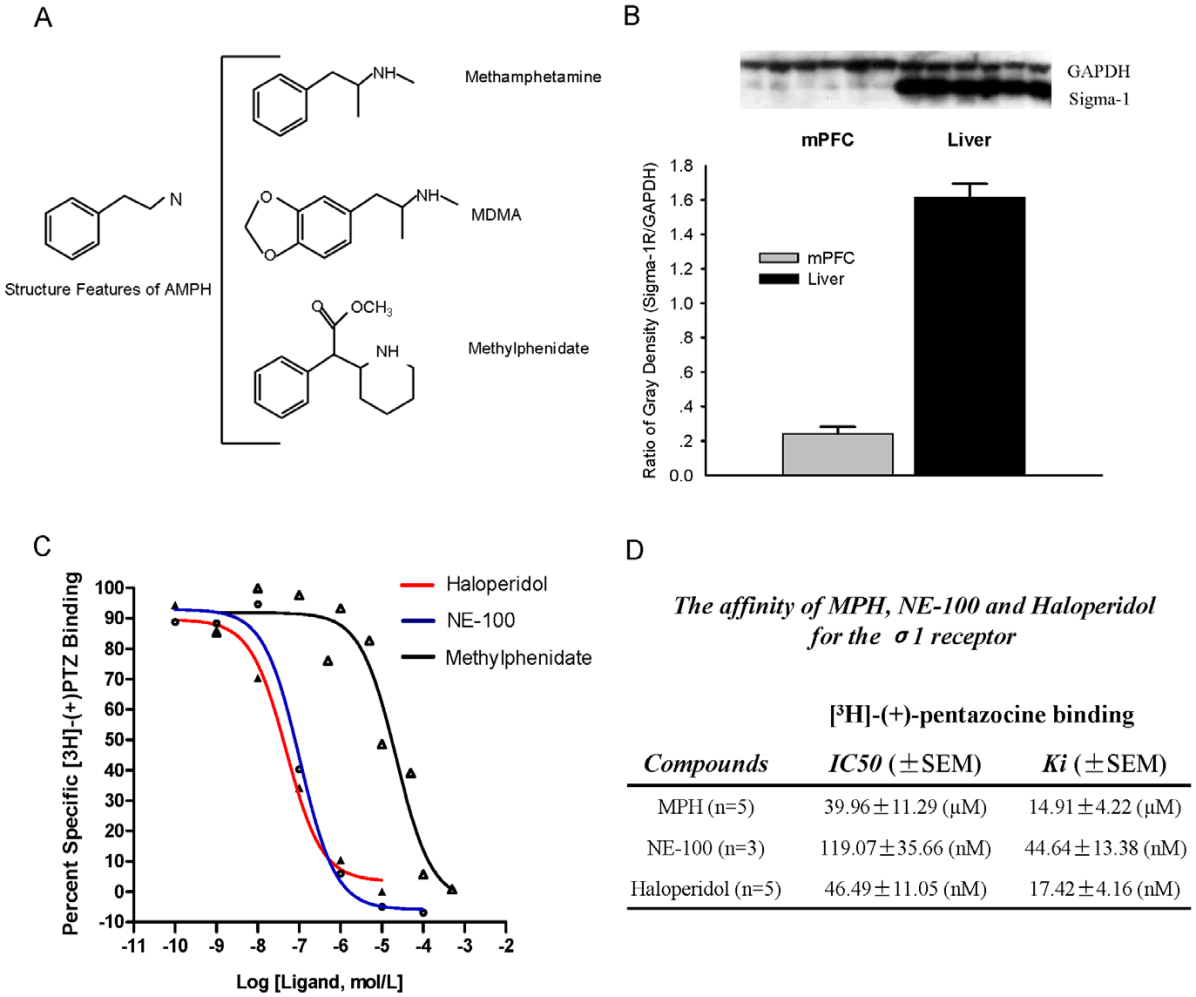
Binding assay of MPH with σ1 receptor. (**A**) MPH has a N-substituted trace amines similar to those of methamphetamine and 3,4-methylenedioxymethamphetamine (MDMA). (**B**) The amount of σ1 receptor in the liver tissue was nearly 8 times of that in the mPFC. (**C**) Competitive binding curves of haloperidol, NE-100, and MPH against [^3^H]-(+)-pentazocine. Haloperidol (10 µM) was used to define non-specific binding. (**D**) Affinities of haloperidol, NE-100 and MPH with σ1 receptor. *IC50* was calculated by nonlinear regression using a sigmoidal function (PRISM, Graphpad, San Diego, CA). Inhibition constants (*Ki*) were calculated using the equation *Ki* = *IC50*/(1+ C/*Kd*), where *Kd* was the equilibrium dissociation constant of σ1 receptor for [^3^H]-(+)-pentazocine (3 nM) in rat liver [54].

It has been shown that σ1 receptor becomes a new binding target for some psychostimulants like cocaine, methamphetamine, and MDMA (3,4-methylenedioxymethamphetamine). Recently, several studies have reported that the σ1 receptor ligand pharmacophore possesses a common N-substituted trace amines [50], [51]. Like methamphetamine and MDMA, MPH also has a similar N-substituted trace amines (Figure 5A), suggesting that MPH could bind with σ1 receptor.

To address this, we conducted competition binding assays. σ1 receptors were labeled in rat liver homogenates, using the radioactive σ1 receptor ligand [^3^H]-(+)-pentazocine (5 nM). Previous study showed that the *Bmax* (maximal number of binding sites) of [^3^H]-(+)-pentazocine for σ1 receptor in the liver (2929 fmol/mg) is nearly 10 times higher than in the brain (280 fmol/mg) [52], [53]. Our western blot experiment also showed the amount of σ1 receptor in the liver is nearly 8 times of that in the mPFC (Ratio of gray density for σ1 receptor/GAPDH in the liver: 1.61±0.08; in the mPFC: 0.24±0.04) (Figure 5B). Thus, we selected liver tissue instead of mPFC tissue to prepare σ1 receptor for binding assays.

**Figure 6:**
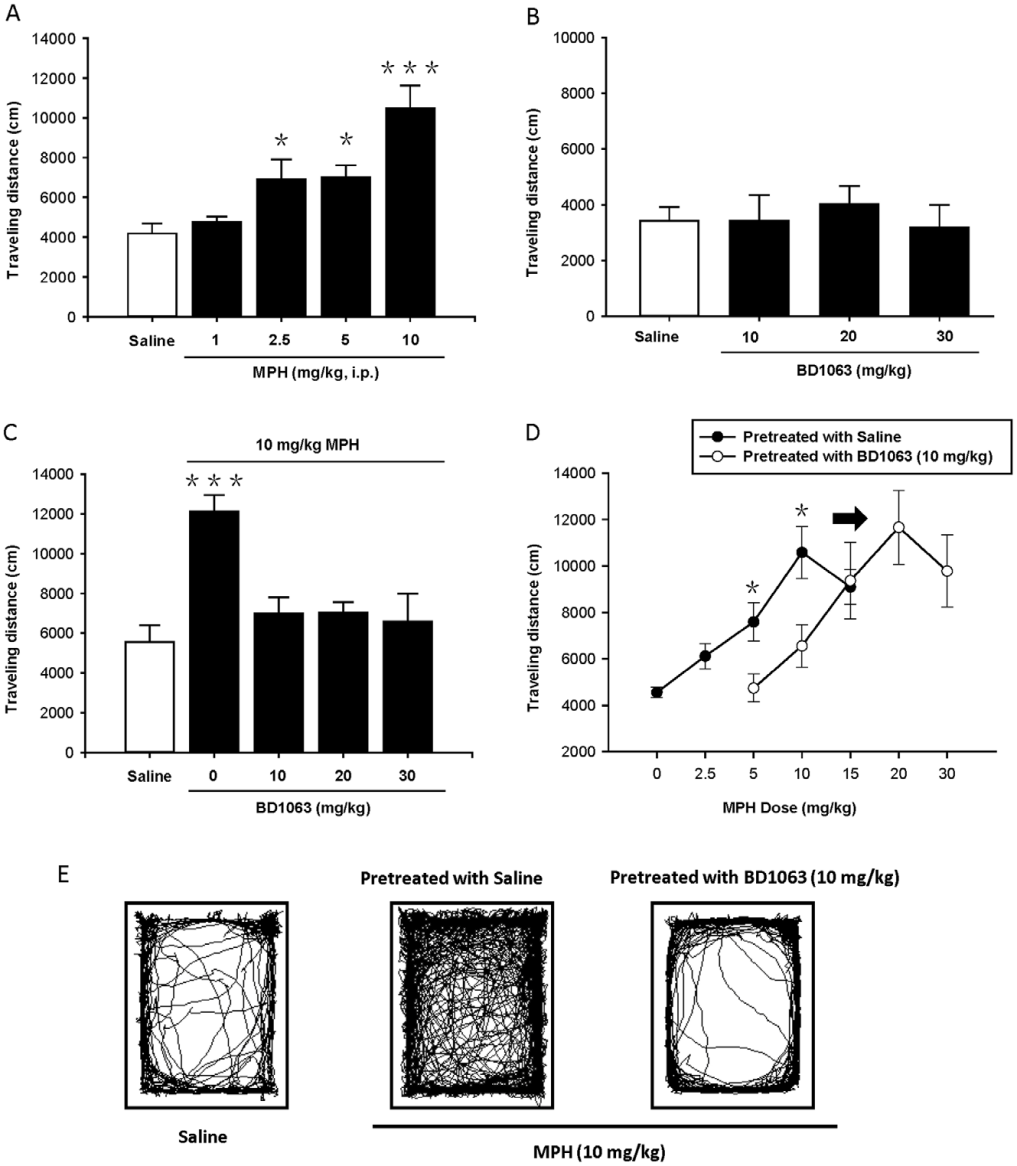
MPH induces locomotor hyperactivity via interaction with σ1 receptor. (**A**) Swiss Webster mice were injected (i.p.) with saline and MPH (1, 2.5, 5 and 10 mg/kg). 30 min later, MPH produced a significant stimulatory effect on locomotor activity in a dose-dependent manner. The horizontal activity was analyzed for 30 min in the open field. *P<0.05 and ***P<0.001 vs. saline, n = 7 for each group, post-hoc Dunnett’s tests. (**B**) BD1063 (10, 20 and 30 mg/kg) itself did not affect basal locomotion of the mice, compared with saline group. n = 7 for each group. No significance. (**C**) Pretreatment with BD1063 (10, 20, and 30 mg/kg) effectively blocked 10 mg/kg MPH-induced locomotor hyperactivity. n = 7 for saline, and n = 6 for other groups. ***P<0.001 vs. saline and other groups, post-hoc LSD multiple comparisons. (**D**) Pretreatment with BD1063 (10 mg/kg) shifted the MPH’s dose-response curves to the right. The mice in the left curve were pretreated with saline, then injected with MPH (0–15 mg/kg). Other group in the right curve was pretreated with BD 1063 (10 mg/kg), then injected with MPH (5–30 mg/kg). MPH with 5 mg/kg and 10 mg/kg groups, *P<0.05 in the absence of BD1063 vs. in the presence of BD1063, n = 7 for each group, post-hoc Student-Newman-Keuls. (**E**) Locomotor activity trace of MPH (10 mg/kg) stimulatory mice pretreated with saline (middle), were significantly different from saline control (left). Pretreated with 10 mg/kg BD1063 (right) effectively blocked MPH’s effect (middle).

Both NE-100 and haloperidol, which are high-affinity σ1 receptor ligands, were used to confirm the reliability of our binding assay system. The competitive binding curves of NE-100, haloperidol and MPH against [^3^H]-(+)-pentazocine were shown in Figure 5C. The inhibition constant (*Ki*) of haloperidol for σ1 receptor was similar with that reported by Klouz et al [54]. Our data showed that MPH could bind with σ1 receptor in a competitive manner (Figure 6C). The *Ki* of MPH for σ1 receptor was 14.91±4.22 µM (Figure 5D).

### σ1 Receptor Involves in MPH-induced Locomotive Hyperactivity

Therefore after, we adapted behavioral pharmacological experiments [35], to test if σ1 receptor involves in MPH-induced locomotor hyperactivity in mice. As described by previous studies, higher doses of MPH lead locmotor hyperactivity in rodents [21], [55], [56]. Indeed as shown in Figure 6A, MPH (2.5, 5, 10 mg/kg, i.p.) produced a stimulatory effect on the locomotor activity of the mice in a dose-dependent manner: saline group (n = 7), 1 mg/kg (P>0.05 vs saline, n = 7, post-hoc Dunnett’s tests), 2.5 mg/kg (P<0.05 vs saline, n = 7), 5 mg/kg (P<0.05 vs saline, n = 7), and 10 mg/kg (P<0.001 vs saline, n = 7). And post-hoc LSD multiple comparisons confirmed that 10 mg/kg group led more evident effect than other groups (F[4], [30] = 11.62, P<0.0001).

In the next antagonism experiments, a selective σ1 receptor antagonist BD1063 [57], was challenged to alter the stimulatory effect of MPH. As indicated by Figure 6B, BD1063 (10, 20, 30 mg/kg) itself did not alter basal locomotor activity of the mice, compared to saline group (n = 7 for each group, no significance). Importantly, we found that pretreatment with BD1063 could effectively block the MPH-induced hyperactivity (Figure 6C and 6E). 10 mg/kg MPH group pretreated with saline (0 mg/kg BD1063, n = 7) was significantly different from saline control (n = 7) and other BD1063 pretreatment groups (F[4], [27] = 10.261, P<0.0001, n = 6 for each group, post-hoc LSD multiple comparisons). Moreover, there was no significance between saline control and MPH pretreated with BD1063 (10, 20, or 30 mg/kg).

Because BD1063 has a higher affinity for σ1 receptor compared with MPH [57], pretreated with BD1063 would pre-occupy σ1 receptors in vivo, and shift the MPH-induced dose-response curves. Indeed, we demonstrated that BD1063 (10 mg/kg) pretreatment elicited an obvious shift to the right in the MPH’s dose-response curves (Figure 6D, see the methods for details). And the locomotor activities of MPH (5 and 10 mg/kg) groups with saline were statistically higher than those pretreated with BD1063 (n = 7 for each group, P<0.05, Student-Newman-Keuls). Taken together, the behavioral pharmacological results provided evidence that higher dose of MPH induces the locomotor hyperactivity via interaction with σ1 receptors.

### MPH Enhances NMDA-R Response via Intracellular Ca^2+^ Dependent PLC/PKC Pathway

It has been documented that physiological effects of σ1 receptor ligands are sensitive to pertussis toxin in vivo and in vitro, suggesting a coupling with cell membrane-bound Gi/o proteins [58]–[62]. However, the cloned σ1 receptor does not have the typical structure for G-protein-coupled receptor (i.e., seven trans-membrane domains). σ1 receptor has at least two subtypes, one metabotropic and the other non-metabotropic [63]. Although current conclusions about the coupling of σ1 receptor to G-protein remain controversial, many studies have shown that σ1 receptor produces physiological effects via calcium-dependent PLC-PKC signaling pathway [47], [64]. Thus, it may be possible that MPH facilitates NMDA-R response in mPFC pyramidal cells through the calcium-dependent PLC-PKC pathway.

**Figure 7:**
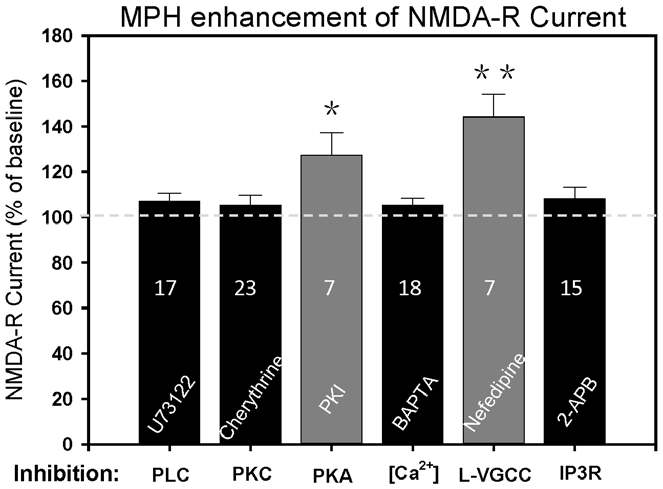
MPH enhancement of NMDA-R current is mediated via intracellular Ca^2+^ dependent PLC/PKC signaling pathway. MPH produced no effect on NMDA-induced current when the PLC inhibitor U73122, the PKC inhibitor chelerythrine, the Ca^2+^ chelating reagent BAPTA, or the IP3 inhibitor 2-APB was administered, but still enhanced the current when the PKA inhibitor fragment 5-24 (PKI5-24) or L-type Ca^2+^ channel blocker nefedipine was applied. NMDA-R currents were recorded in the presence of CNQX, TTX and BMI, with holding potential of -40 mV. Shown in figure are the normalized histograms for MPH effects in the presence of U73122, chelerythrine, PKI5-24, BAPTA, nefedipine and 2-APB. *P<0.05 vs. control. **P<0.01 vs. control, paired t-test. Numbers mean the cell recorded.

To address this possibility, we first pre-incubated brain slices with the PLC inhibitor U73122 (20 µM) for at least 60 min. During recordings, U73122 was added into internal solution (5 µM). In some cells, U73122 was also bath applied (10 µM). As shown in Figure 7, MPH failed to enhance NMDA-induced currents in the presence of U73122 (106.9±3.7% of the baseline, n = 17, P>0.05), indicating that PLC is a critical step that mediates the facilitation effect of MPH.

It has been reported that stimulation of σ1 receptor facilitates NMDA-induced pain via PKC- and PKA-dependent phosphorylation of NMDA-R NR1 subunit in mouse spinal cord [65]. Thus, we tested if PKC and/or PKA play a role in MPH facilitation of NMDA-R response. The PKC inhibitor chelerythrine (20 µM) or the PKA inhibitor fragment 5–24 (PKI5-24; 1 µM) was added into internal pipette solution. In some cells, chelerythrine was also bath applied (10 µM). We found that the MPH facilitation was blocked in the presence of chelerythrine (105.3±4.3% of the baseline, n = 23, P>0.05), but still was there in the presence of PKI5-24 (127.3±9.9% of the baseline, n = 7, P<0.05) (Figure 7). These results indicated that MPH enhancement of NMDA-R response involves PKC but not PKA.

Activated PLC cleaves phosphoinositol-4,5-bisphosphate (PIP2) into 1,2-diacylglycerol (DAG) and inositol-1,4,5-trisphosphate (IP3) [66]. It is known that activation of a conventional PKC requires intracellular Ca^2+^ and DAG [67]–[69]. Thus, we next tested if the facilitation effect of MPH requires intracellular Ca^2+^. To do this, we added the highly selective calcium chelating reagent BAPTA (10 mM) into internal pipette solution. As shown in Figure 7, MPH did not enhance the NMDA-induced current in the presence of BAPTA (101.8±3.6% of the baseline, n = 18, P>0.05), indicating that elevation of intracellular Ca^2+^ level is essential for the MPH facilitation.

It is reported that σ1 receptor regulates Ca^2+^ concentration via extracellular Ca^2+^ influx (via voltage-sensitive L-type Ca^2+^ channels) and/or intracellular Ca^2+^ mobilization from endoplasmic stores (via IP3 receptors) [44]. We then investigated MPH effect when the L-type calcium channel blocker nefedipine (10 µM) or the membrane-permeable IP3 inhibitor 2-APB (60 µM) was continuously administered in bath. MPH still enhanced the NMDA-induced current in the presence of nefedipine (144.1±9.9% of the baseline, n = 7, P<0.01), whereas it failed to facilitate the current in the presence of 2-APB (108.1±5.1% of the baseline, n = 15, P>0.05) (Figure 7). Thus, MPH facilitation of NMDA-R response requires IP3-dependent release of Ca^2+^ from intracellular Ca^2+^ store.

## Discussion

Antagonism of NMDA-receptor (NMDA-R) has been shown to prevent acute and chronic MPH-leading behavioral sensitization like locomotor hyperactivity [55], [70], indicating that NMDA-R plays an crucial role in MPH-induced effects. In such behavioral sensitization animals, MPH indeed facilitates the postsynaptic NMDA-R mediated EPSC at PFC-ventral tegmental area (VTA) glutamatergic synapses [71]. In line with this, our results demonstrate for the first time that bath application of MPH enhances, via postsynaptic σ1 receptors, NMDA-R mediated excitatory synaptic transmission in pyramidal cells of the medial prefrontal cortex (mPFC) of rats. Further receptor binding assays and behavioral pharmacological studies confirm that MPH leads the locomotor hyperactivity in rodents, via interaction with the σ1 receptor, which implies the underlying receptor mechanism for MPH induced effects like addictive potential.

### Postsynaptic Action of MPH

MPH would facilitate excitatory synaptic transmission, mainly through strengthening catecholaminergic synaptic transmission. For example, MPH infusion into amygdala facilitates postsynaptic AMPA-R mediated current at cortico-amygdala synapses, via indirect stimulation on D1 receptor [72]. However, MPH may regulate non-NMDA- and NMDA-R mediated excitatory synaptic transmission through different mechanisms. As shown by Prieto-Gomez et al, higher doses of MPH produce facilitating effects on NMDA-R mediated EPSC but not non-NMDA-R mediated EPSC [71]. Indeed, our results indicated that MPH facilitation of non-NMDA-R mediated synaptic transmission may involve a catecholamine-dependent presynaptic mechanism. On the other hand, there exists a catecholamine-independent mechanism, a postsynaptic action for MPH facilitation of NMDA-R mediated synaptic transmission. Additionally, we showed that MPH could enhance the NMDA-R current induced by pressure-administered NMDA directly onto cellular soma. Thus these findings demonstrated that MPH acts via postsynaptic mechanism to regulate NMDA-R mediated excitatory synaptic transmission.

### Role of σ1 Receptor in MPH Facilitation

As shown by previous studies, DA selective reuptake blockers (GBR-12783 and GBR12909) [19], [73] or another selective NE reuptake inhibitor desipramine [19], could not completely mimick MPH-induced effects, suggesting that inhibition of DA/NE transporters alone could not explain the mechanism of MPH. There might be other receptors directly involving in the multiple effects of MPH. Although D1 receptor stimulation also potentiates NMDA-R mediated synaptic transmission in PFC [46], [74], [75], the present study excludes the direct involvement of D1 receptor in MPH’s facilitation of NMDA-R activity. We reason that the non-involvement of D1 receptor is possibly due to our experiments conducted in pharmacological isolated condition. Thus, one may debate that MPH possibly modulate NMDA-R activity via both D1 and σ1 receptors in physiological situation. Interestingly, Navarro et al recently provided first evidence for coexistence of σ1-D1 receptor heteromerization in the brain. And it has been shown that stimulation on σ1 receptor or co-stimulation on σ1-D1 receptors could robustly potentiate D1 receptor-mediated effects [76], [77].

**Figure 8:**
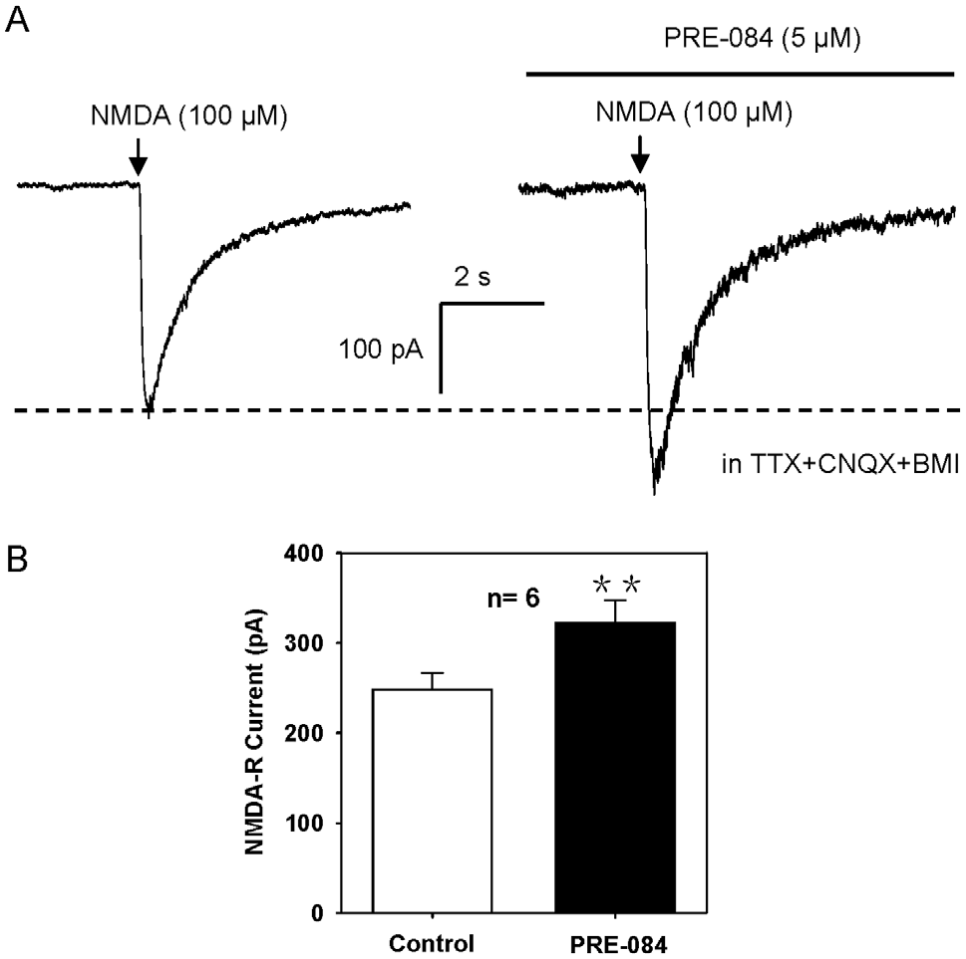
PRE-084 enhances NMDA-induced current. The specific σ1 receptor agonist PRE-084 (5 µM) enhanced NMDA-induced current, as MPH did. Recordings of NMDA-R current were performed in the presence of CNQX (20 µM), TTX (1 µM) and BMI (20 µM), with holding potential of −40 mV. **P<0.01 vs. control, n = 6, paired t-test.

As a new receptor target for cocaine and amphetamine, σ1 receptor plays an important role in stimulants-induced drug sensitization development and locomotor stimulation [32], [34], [35], [78]. Consistently in the present study, we provide pharmacological evidence that MPH interacts with σ1 receptor, and induces locomotor hyperactivity. Moreover, activation of σ1 receptor alone could powerfully potentiate NMDA-R mediated responses [43], [79]–[82]. For example in rat hippocampus, activation of σ1 receptor increases NMDA-induced current and long-term plasticity, confirming the important role of σ1 receptor in potentiating the excitatory synaptic transmission [48]. To mimick the MPH enhancement of NMDA-R response in PFC, we applied a selective σ1 receptor agonist, PRE-084, and found that σ1 receptor stimulation facilitated NMDA-induced current (130.5±9.2% of the baseline, n = 6, P<0.01, paired t-test; Figure 8). Taken together, we show a new clue that MPH exerts action on σ1 receptor to potentiate NMDA-R mediated synaptic transmission in PFC, which may underlie the mechanism for MPH-induced locomotor hyperactivity.

However, our receptor binding assays showed that the Ki of MPH for σ1 receptor is at around 10 µM, which is not at a comparable level of its affinity for DA/NE transporters (around 0.1 µM) [83]. Indeed in the VTA slices pretreated with MPH, the doses of MPH (from 2.5 to 20 µM) increase the mEPSC frequency, but only higher doses (more than 10 µM) could enhance NMDA-R mediated EPSC. Consistently in our study, MPH (50 µM) facilitating both non-NMDA-R and NMDA-R mediated EPSC is at a higher level. Thus, possibly due to differentially modulation of DA/NE systems or/and σ1 receptor, MPH may exert stimulation on excitatory synaptic transmission in a dose-dependent manner. In addition, Swanson and Volkow showed that even at clinical doses, MPH administered intravenously, not orally, would produce reinforcing effects like euphoria while binding more than 60% DA transporters [84], suggesting that both doses and rapid escalation in serum are crucial for MPH induced addiction and psychiatric side effects.

### Intracellular Pathway for MPH Facilitation

Emerging evidence have shown that stimulants (cocaine, amphetamine and MPH) profoundly alter the phosphorylation status of NMDA-R and/or AMPA-R in the central nervous system, via PKC and/or PKA signaling pathways [85]–[87]. For example, administration of 10–20 mg/kg MPH could increase the phophorylation of GluR1 subunit for AMPA-R in the PFC in vivo, via activation of cAMP/PKA pathway through β1-adrenergic receptor [86]. Through such phosphorylation of NMDA-R and AMPA-R, the stimulants may shape the synaptic plasticity related to additive properties of drugs.

cAMP/PKA signaling pathway, indicated as a therapeutic target for memory disorders, plays a key role in memory processes. Proper modulation of this signaling pathway may improve PFC function, whereas excessive stimulation may impair the PFC cognitive functions, like working memory [1], [88]. Similarly, although PKC plays an important role in learning and memory [89], overactivity of PKC signaling impairs the PFC regulation of working memory [90]. In addition, Brennan et al reported that blockage of IP3 receptor mediated PKC signaling enhances working memory [91], suggesting that dysregulation of PKC signaling by medication or mental disorders would result in dysfunction of the PFC. Taken together, these data elucidate a unique perspective toward understanding the molecular basis of MPH’s therapeutic or/and impairing actions.

σ1 receptor stimulation modulates Ca^2+^ entry through plasma membrane and intracellular Ca^2+^ release [43], [44], which may subsequently activate Ca^2+^ dependent PKA or/and PKC signaling pathways. For example, Kim et al reported that σ1 receptor facilitates NMDA-induced effects via both PKC- and PKA-dependent signaling pathways [65]. And Fu et al showed that σ1 receptor stimulation on PKC signaling cascade amplifies the D1 receptor mediated PKA signaling in PFC [77]. However recently, studies mainly focus on the key role of PLC/PKC signaling in σ1 receptor leading effects [47], [64]. Consistently in the present study, we show that Ca^2+^ dependent PLC/PKC signaling pathway is important for MPH’s direct modulation of NMDA-R activity.

In the PLC/PKC pathway, activated PLC cleaves PIP2 into DG and IP3 [66]. Then under IP3 induced intracellular Ca^2+^ release, DG kinase (DGK) as a lipid kinase, binds to PKC to activate more intracellular signaling pathways [69]. Interestingly in a recent report, mice mutant with one subtype of the DGK, DGKβ, have been shown to express various dysfunctions similar with ADHD symptoms. In the open field test, these DGKβ KO mice could not display locomotor hyperactivity elicited by MPH (30 mg/kg, i.p.) like WT mice, which results from the dysregulation of ERK phosphorylation [92]. Thus, the study indicates an important linkage among the PLC/DG/PKC signaling pathway, the stimulating actions of MPH and ADHD pathology.

**Figure 9:**
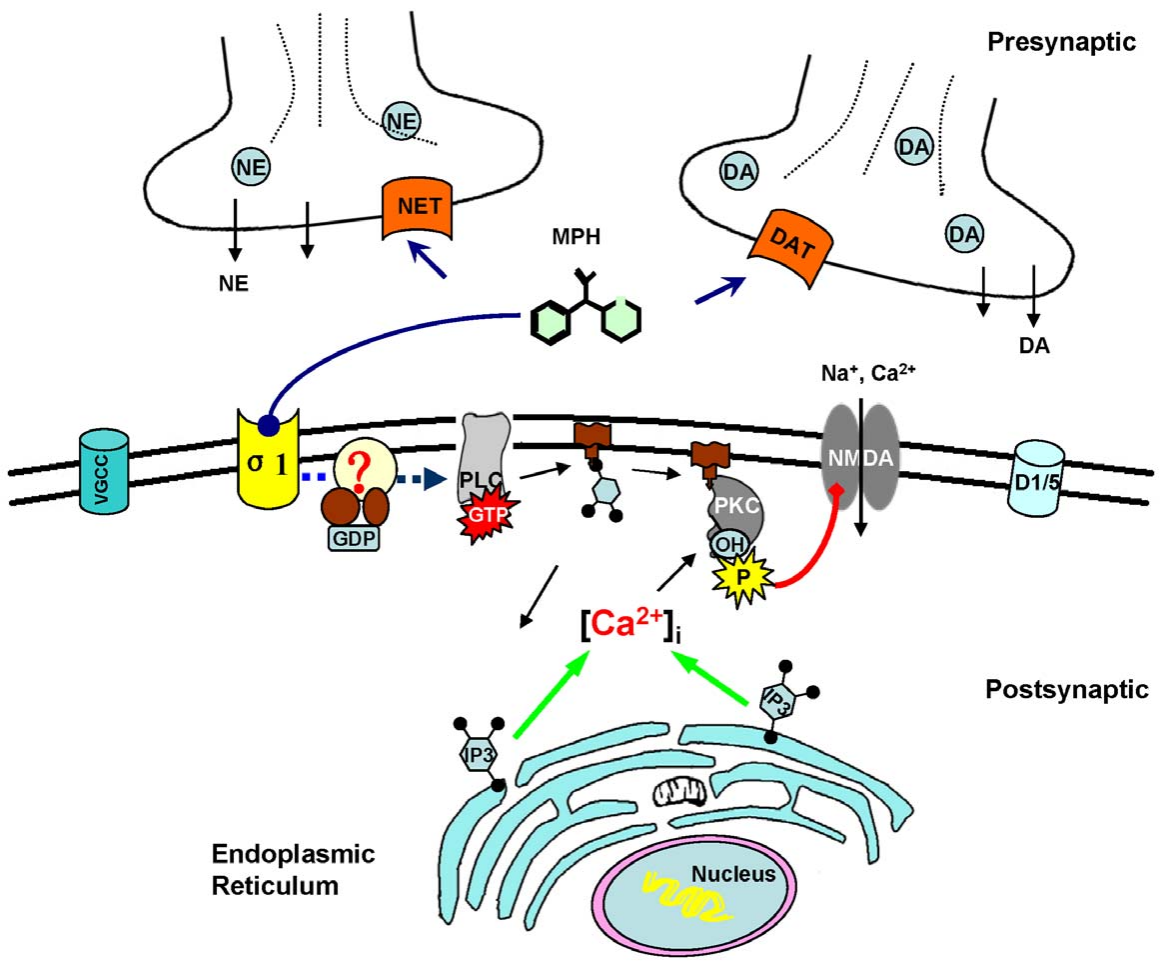
A novel mechanism for the MPH enhancement of NMDA-R activity. As a blocker of DA/NE transporters, MPH increases the DA/NE level in the synaptic cleft. In addition, MPH at higher dose could potentiate NMDA-R mediated exciatatory synaptic transmission, via a catecholamine-independent mechanism. Through action at postsynaptic σ1 receptor, MPH activates PLC, which subsequently cleaves PIP2 into DG and IP3. Under IP3-induced intracellular Ca^2+^ release, PKC activated by DG may result in the phosphorylation of NMDA receptors, and enhancement of NMDA-R activity.

Taken these evidence together, to illustrate the intracellular pathway in the MPH’s facilitation of NMDA-R responses in pyramidal cells of PFC, we draw a figure showing that: 1) as a DA/NE transporter blocker, MPH would first strengthen catecholaminergic transmission; 2) via a catecholamine-independent mechanism, MPH at a higher level could act at postsynaptic σ1 receptor; 3) subsequent activated PLC/IP3/PKC signaling pathway may result in the phosphorylation of NMDA receptors and thus enhance NMDA-R activity (Figure 9).

### Potential Physiological Significance

Higher doses of MPH administered acutely or chronically have been reported to induce addiction [1], [21]–[23], which may result from excessive stimulation on σ1 receptors according to previous reports on other stimulants [30], [33], [35]. Moreover in ADHD patients, repeated or long-term treatment with MPH produces psychiatric adverse effects like depression [29], [93]. For example, A 7-year-old boy with ADHD after raising the dose from minimal, developed clinical signs of depression, which could be withdrawed if the treatment was ceased [29]. Coincidently, long time use of MPH from preadolescence leads to anxiety and depression-like behaviors in adult [28]. In the MPH-induced depressive-like animals, fluoxetine as both selective serotonin reuptake inhibitor and σ1 receptor agonist [42], could alter MPH-leading effects [27], [28], suggesting that fluoxetine and MPH may share the same σ1 receptor mechanism. Thus, these evidence provide a perspective use of σ1 receptor ligands to prevent the MPH-induced addiction and depressive side effects in clinic.

As NMDA-R is essential for synaptic plasticity and learning/memory function [94], [95], σ1 receptor regulation of NMDA-R function is proposed to be involved in learning and memory [42], [96]. Importantly, σ1 receptor is highly distributed in the PFC, striatum and hippocampus, which are areas important for learning and memory [42], [97]. In hippocampus, activation of σ1 receptor by neuroactive steroids and non-steroidal sigma ligands, potentiates NMDA-R mediated responses [48], [79]–[81], [98]–[100]. And such action recruits Ca^2+^-dependent PKC signaling pathway [47]. In striatum, σ1 receptor agonists could regulate NMDA-stimulated dopamine release, also possibly requiring PKC signaling [101], [102]. Thus, it remains to be demonstrated in future if σ1 receptor possibly involves in MPH’s therapeutic actions like learning and memory improvement.

In clinical studies, σ1 receptor agonists have been shown to improve cognitive impairment and neuropsychiatric diseases symptoms like depression, stress, anxiety and senile dementia [42], [96], [103]. Thus, σ1 receptor is a potential therapeutic target for those psychiatric disorders. Given that MPH is a ligand for σ1 receptor, it would be possible that MPH might be used as a medication for these psychiatric disorders. Interestingly, MPH has already been treated with pathological depression, dementia and other psychiatric disorders [104]–[106].

In conclusion, the present study elucidates the role of σ1 receptor and PLC/PKC signaling pathway involving in MPH facilitation of NMDA-R mediated synaptic transmission in pyramidal cells of the medial prefrontal cortex, which possibly implies the underlying mechanism for the MPH-induced addictive potential and other psychiatric adverse effects.

## Materials and Methods

### Ethics Statement

The present study was strictly in compliance with Guide for the Care and Use of Laboratory Animals of the National Institutes of Health. All the experimental protocols used were approved by the Committee on the Ethics of Animal Experiments of the Fudan University (Permit Number: 2007–0002). All surgery was performed under deeply anaesthesia with isoflurane or sodium pentobarbital, and all efforts were made to minimize the suffering of animals.

### Brain Slice Preparation

Spraque-Dawley rats (14∼25 days) were purchased from the Laboratory Animal Center, Fudan University Shanghai Medical School. Brain slices were prepared according to the procedures described previously [107]. In brief, rats were deeply anaesthetized with isoflurane. Brains were quickly removed (within 1 min), submerged in ice-cold artificial cerebrospinal fluid (ACSF) containing (in mM): 119 NaCl, 2.5 KCl, 1 CaCl_2_, 3 MgSO_4_, 1 NaH_2_PO_4_, 26.2 NaHCO_3_, 11 glucose, and saturated with 95% O_2_∼5% CO_2_. Coronal bilateral slices (350∼400 µm in thickness) containing the mPFC were then cut on a Vibroslice (MA752, Campden Instruments, US). Three or four slices from each hemisphere were transferred to an oxygenated ACSF incubation bath, and incubated for at least 1 h before recording. The perfusion ACSF was delivered with a pump (Peri-Star 291, World Precision Instruments, USA) at a rate of 2∼3 ml/min. In the perfusion ACSF, the concentration of CaCl_2_ was 2.5 mM and that of MgCl_2_ was 1.5 mM. All experiments were performed at room temperature (23∼25°C).

### Identification of Pyramidal Cells

A slice was viewed with an upright microscope (Axioskop FS mot, Zeiss, Germany) equipped with infrared-differential interference contrast (IR-DIC) optics. Slice image was detected with an Infra-Red-sensitive CCD (C2400-79H, Hamamatsu, Japan) and displayed on a black-white video monitor. Pyramidal cells in layer V∼VI of the mPFC could be recognizable via a 40× water-immersion lens. A typical pyramidal cell exhibited a firing pattern with spike frequency adaptation in response to a depolarization current. In some cells examined, Lucifer yellow was added into internal pipette solution at a concentration of 0.05% (W/V). The dye could enter into the body of a patched cell through diffusion. At the end of an experiment, the slice was fixed in 4% paraformaldehyde. The cell’s morphology was further confirmed under a fluorescence microscope (DMRXA, Leica, Germany).

### Chemical Drugs

Purchased from the Sigma Chemical Company (Sigma, St. Louis, MO, USA) were ATP.Mg^2+^, bicuculline methiodide (BMI), glutamate, GTP.Na^3+^, haloperidol, K^+^ gluconate, methylphenidate hydrochloride (MPH), nefedipine, reserpine, yohimbine hydrochloride, 2-aminoethyl diphenylborinate (2-APB), 1-pyrrolidinylethyl 3,4-dichlorophenylacetate oxalate salt (AC915), DL-2-amino-5-phophonovaleric acid (AP-5), (1,2-bis(2-aminophenoxy)-ethane-N,N,N’,N’-tetraacetic Acid (BAPTA), 6-cyano-7-nitroquinoxaline-2, 3-dione (CNQX), ethyleneglycolbis aminoethyl ethertetra-acetate (EGTA), N-[2-hydroxyethyl] piperazine-N’-[2-ethanesulfonic acid] (HEPES), N-methyl-D-aspartic acid (NMDA), 2-(4-morpholino)ethyl-1-phenylcyclohexane-1-carboxylate hydrochloride (PRE-084), and 1-[6-[((17β)-3-methyl-N-[7-(1-pyrrolidinyl)-1-oxaspiro[4.5]dec-8-yl]-benzeneacetamide (U73122). Chelerythrine chloride, 1-[2-(3,4-dichlorophenyl)ethyl]-4-methylpiperazine dihydrochloride (BD1063), protein kinase A inhibitor fragment 5–24 (PKI5-24), and SCH 39166 hydrobromide were purchased from the Tocris Cookson Ltd. (Ellisville, Missouri, USA). Tetrodotoxin (TTX) was purchased from the Research Institute of Aquatic Products, Hebei Province, China. [^3^H]-(+)-pentazocine (28 Ci/mmol, #NET1056) was purchased from the Perkin-Elmer Inc. (Boston, MA, USA).

Most of the drugs were dissolved as stocks in ultra-pure deionized water produced by an untrapure system (Millipore Q-Gard 1, Billerica, MA) except for haloperidol and nefedipine, which were dissolved in ethanol as stocks. Most of stock solutions such as TTX and BMI were kept at 0∼4°C (not more than 7 days prior to use). Other stock solutions like AP-5, CNQX and internal pipette solution were stored at −20°C. PKI5-24 stock was stored in frozen aliquots at −80°C. All stocks were diluted with ACSF before application.

### Patch-clamp Recordings

Whole-cell recordings were made in layer V∼VI pyramidal cells in the mPFC. Patch pipettes (3∼7 MΩ) were fabricated from borosilicate tubing (1.5 mm in outside diameter and 0.86 mm in inside diameter; Sutter Instruments, Navato, CA, USA), using a horizontal microelectrode puller (P-97, Sutter Instruments). The internal pipette solution contained (in mM): 150 K^+^ gluconate, 0.4 EGTA, 8 NaCl, 2 ATP.Mg, 0.1 GTP.Na^+^_3_ and 10 HEPES, with pH value adjusted to 7.2∼7.4 by KOH, and had an osmolarity of 290∼320 mOsm. Voltage and current signals in current- and voltage-clamp modes were recorded with a HEKA EPC-9 amplifier (Heka, Germany), which was connected to a Digidata interface (Molecular Devices, Union City, CA). The electrophysiological data were digitized and stored on disks using Pulse software (Heka, Lambrecht, Germany). Recordings of resting membrane potential and action potential were performed under the current-clamp mode. The mode was then shifted to the voltage-clamp mode for recordings of eEPSC, glutamate-induced non-NMDA-R currents and NMDA-induced currents. For individual cells, the series resistance (Rs) was monitored at regular intervals throughout recording, which was among 10∼20 MΩ. Data were discarded if the Rs of a recorded cell changed by 15%.

For recordings of eEPSC, a custom-made bipolar stimulation electrode was positioned 200 µm subjacent to a recorded cell. Current pulses (50∼100 µA in amplitude, 100 µsec in duration, and 0.033 Hz in frequency) were generated by Master-8 (A.M.P. Instruments Ltd., Jerusalem, Israel). For recordings of glutamate-induced non-NMDA-R current or NMDA-induced current, glutamate or NMDA (100 µM; dissolved in ACSF) was puff delivered to the soma of a patched cell from a distance of ∼20 µm, using a patch pipette (8∼10 µm in tip diameter). Pressure application was controlled by a pneumatic picopump (PV820, World Precision Instruments, USA), with an inter-pulse interval of at least 1 min.

### Drug Administration

As ionotropic glutamate receptors, including NMDA-R and non-NMDA-R, are present not only in pyramidal cells but also in GABAergic interneurons, it is necessary to block any possible and indirect influence from GABAergic interneurons. Hence, the GABAergic antagonist BMI was continuously applied during recordings. TTX (1 µM) was bath-applied to block voltage-activated sodium channels and therefore eliminate spontaneous action potentials at pre-synaptic terminals that would trigger glutamate releases. For recordings of eEPSC, the NMDA-R antagonist AP-5 (50 µM) or the non-NMDA-R antagonist CNQX (20 µM) was bath-applied to isolate the current mediated by non-NMDA receptor or NMDA receptor. For recordings of glutamate-induced non-NMDA-R current, AP-5 (50 µM) and TTX (1 µM) were continuously co-applied in the perfusate, whereas CNQX (20 µM) and TTX (1 µM) were co-administered for recordings of NMDA-induced current. MPH (1, 10 or 50 µM) was bath applied and limited to one cell per slice.

Other drugs were bath applied continuously in the perfusate before and during application of MPH, including haloperidol (1 µM, a potent σ1 receptor antagonist), AC915 (1 µM, a potent and selective σ1 receptor antagonist), yohimbine (1 µM, an a2 receptor antagonist), nefedipine (10 µM, a L-type calcium channel antagonist), SCH39166 (1 µM, a D1/5 receptor antagonist), 2-APB (60 µM, a membrane-permeable inhibitor of intracellular IP3-induced calcium release), U73122 (10 µM, an inhibitor of the coupling of G protein-PLC activation), and chelerythrine (10 µM, a potent and cell-permeable inhibitor of PKC). In the experiments with PLC inhibition, brain slices were first pre-incubated with U73122 (20 µM) for at least 60 min. To inhibit the activity of postsynaptic intracellular PLC, PKC or PKA, U73122 (5 µM), chelerythrine (20 µM) or PKI5-24 (1 µM) was added into the internal pipette solution. To chelate intracellular Ca^2+^, the selective calcium chelating reagent BAPTA (10 mM) was added into the internal pipette solution. To examine the effect of σ1 receptor activation on NMDA-R response, PRE-084 (5 µM, a potent and selective σ1 receptors agonist) was bath applied.

### Depletion of Catecholamine

In this experiment, we used reserpine, an inhibitor of the vesicular monoamine transporter, to deplete presynaptic catecholamine. Rats were pre-treated with reserpine (1.5 mg/kg, i.p.) 2 hours before anesthesia. Slices were kept in ASCF containing reserpine (10∼20 µM) for more than 30 min. The concentrations of NE and DA in reserpine-treated slices were detected with fluorescence spectrophotometric method [108].

Briefly, slices (<0.5 g) were first homogenized for 5 min in butanol (5 ml) containing HCl (0.01 M). The homogenate was centrifuged at 3000 g for 10 min. The supernatant (2 ml) was added with phosphate buffer (0.1 M, 1.5 ml; pH 6.5), oscillated for 10 min, and centrifuged at 3000 g for 10 min. Then, the subnatant (1 ml) was added with EDTA (0.1 M, 0.4 ml) and iodine (0.1 M, 0.2 ml). After oscillated for 2 min at 30°C, the liquor was added with Na_2_SO_3_ (0.2 M, 0.4 ml), and oscillated once again for 2 min at 30°C. Next, the liquor was acidified to pH 4.4∼4.8 with 0.5 ml HAc (6 M), and heated in an oven at 100°C for 2 min. The liquor tubes were ice cooled, and NE fluorescence was measured spectrophotometrically at 385∼485 nm, by using a spectrophotofluorometer (DU 7500, Beckman Coulter, USA) at 25°C. In order to develop DA fluorescence, the liquor tubes were placed back to the oven and heated at 100°C for 15 min. After the tubes were ice cooled, DA fluorescence was measured spectrophotometrically at 310∼390 nm at 4°C.

### Western Blot Analysis

mPFC and liver tissues were collected from 6 rats for isolation of protein. Tissue samples were homogenized in an ice-cold lysis buffer (Beyotime Institute of Biotechnology, China; #P0013) with 1 mM phenylmethanesulfonyl fluoride (PMSF) and 0.5% protease inhibitors (Protease Inhibitor Cocktail; Roche Diagnostics Corporation, USA). The homogenates were centrifuged at 10,000 rpm for 10 min at 4°C. An aliquot of the supernatant was taken to measure protein concentration, using Micro BCA protein assay reagent kit (Pierce Protein Research Products, Thermo Scientific, USA; #23235). The remaining supernatant was stored at −80°C.

The protein was quantified as 30 µg per lane, and mixed with a volume of ultra-pure deionized water and 2× sample buffer. The samples were boiled for 5 min, and loaded to 8% sodium dodecylsulfate polyacrylamide gel electrophoresis (SDS-PAGE). The samples were then transferred electrophoretically onto polyvinylidene fluoride (PVDF) membranes (Roche Diagnostics Corporation, USA), using an electrophoresis system and a mini trans-blot electrophoretic transfer system (Bio-Rad Laboratories, Inc., USA). To attenuate non-specific staining, the membranes were blocked for 2 hours at room temperature with 5% non-fat dry milk in Tris-buffered saline with Tween (TBST; 20 mM Tris-HCl, 137 mM NaCl and 0.1% Tween-20; pH 7.6), and then incubated overnight with the primary antibody against σ1 receptor at 4°C (dilution 1∶100; Santa Cruz Biotechnology, Inc., USA; #sc-22948). The blots were washed three times (10 min each time) in TBST, and incubated for 2 hours with horseradish peroxidase (HRP)-conjugated donkey anti-goat IgG (dilution1∶7000; Jackson ImmunoResearch Laboratories, Inc., USA; #705-035-147). The membranes were again washed three times (10 min each time) in TBST.

Immunoreactivity signals were detected using the enhanced chemiluminescent substrate (SuperSignal West Femto Maximum Sensitivity Substrate; Pierce Protein Research Products, Thermo Scientific, USA; #34095). X-ray films were exposed to the membranes for seconds, and developed for visualization of the immunoreactivity bands. To estimate the molecular weights of aimed proteins, a pre-stained marker (Tiangen Biotech Company, Ltd., China; #MP205) was used. Photoshop software was used to determine the difference of gray density level. In the experiments, GAPDH (dilution 1∶60000; Cell Signaling Technology, USA; #3683) was used as control.

### Receptor Binding Assays

Samples of liver cellular membrane were prepared from Spraque-Dawley rats (150∼200 g), using the procedures described previously [109]. Liver tissues were quickly processed and homogenized with a glass homogenizer (8∼10 up and down strokes) in a volume (5 ml/g) of cold buffer (0.32 M sucrose in 50 mM Tris, pH 7.4). The tissues were then homogenized with a Tissue-Tearor (Biospec Products, Inc., USA), and centrifuged at 1200 g for 10 min at 4°C. The supernatant was centrifuged at 25000 g for 20 min at 4°C. The pellet was then suspended in a volume (5 ml/g) of 50 mM Tris (pH 7.4) and centrifuged at 25000 g for 20 min at 4°C, and such procedure was repeated twice. The final pellet was suspended in a volume (5 ml/g) of the Tris buffer and stored at −80°C. Protein concentration in the membrane preparation was about 2.0∼2.5 mg/ml, which was determined using the previously-described procedures [110].

The affinity of MPH for σ1 receptor was determined by competitive binding assay. Membrane preparation (250∼400 µg protein) in duplicated tubes were incubated with 5 nM [^3^H]-(+)-pentazocine (28 Ci/mmol) and different concentrations of MPH, NE-100 or haloperidol for 180 min at 30°C in a total volume of 200 µl binding buffer (50 mM Tris, 4 mM MgCl_2_, pH 7.4). And non-specific binding was measured in the presence of 10 µM haloperidol. The reaction was stopped by rapid filtration through Whatman GF/B glass fiber filter, and the filters was washed with ice-cold buffer (50 mM Tris, 5 mM EDTA, pH 7.4) using a Brandel 24-well cell harvester. The filters were then dried at 80°C for 30 min in an oven. Scintillation cocktail was added, and the radioactivity of the filters was determined in a MicroBeta liquid scintillation counter.

The *IC50* value, that is, the concentration of MPH, NE-100 or haloperidol that causes 50% inhibition of the specific binding of [^3^H]-(+)-pentazocine, was calculated by nonlinear regression using a sigmoidal function (PRISM, Graphpad, San Diego, CA). Inhibition constants (*Ki*) value was calculated using the equation *Ki* = *IC50*/(1+ C/*Kd*) [111], where *Kd* was the equilibrium dissociation constant of σ1 receptor for [^3^H]-(+)-pentazocine (3 nM) in the liver tissue of rats [54]. Experiments with MPH and haloperidol were performed in duplicates and repeated four times (n = 5), and those with NE-100 were repeated three times (n = 3).

### Locomotor Activity

The behavioral experiments on Swiss Webster mice were conducted in a 48×40×30 cm open field. To decrease the anxiety or stress, all the mice were habituated in the box 15–30 min before drug application. After the drug application, the mice were put back to cages for 30 min rests. During the tests, the locomotor activity was recorded for 30 min by a digital camera, analyzed by a “tracking-mouse” software programmed by Dr. Jiyun Peng in the lab, and converted to distance traveled (cm). First, groups of mice were injected intraperitoneally (i.p.) with saline and MPH (1, 2.5, 5, 10 mg/kg). Then the most evident dose of MPH (10 mg/kg) was used in the next experiments. The selective σ1 receptor antagonist BD1063 (Tocris, UK) was dissolved in saline, and used according to previous studies [35]. Before the antagonism experiments, three doses of BD1063 (10, 20, 30 mg/kg, i.p.) were compared to saline control, to check if the drug itself would influence the locomotor activity. Then, four doses of BD1063 (0, 10, 20, 30 mg/kg; saline as 0 mg/kg) were used to antagonism the 10 mg/kg MPH-induced the hyperactivity, which could be compared with saline control. In the end, 2 big groups of mice were selected for the MPH dose-response curves. The first group was pretreated with saline 15 min before administered with MPH (0, 2.5, 5, 10, 15 mg/kg; saline as 0 mg/kg). The second group was pretreated with 10 mg/kg BD1063 15 min before administered with MPH (5, 10, 15, 20, 30 mg/kg). After MPH injection, additional 30 min rests were taken before tests.

### Data Analysis

All data in the figures are expressed as means±SEM. The eEPSC traces in the figures were the average of 10∼15 consecutive responses. The traces for non-NMDA- and NMDA-R currents were the average of 5∼10 consecutive responses. Off-line analysis was performed using Igor Pro (Wave Metrics, USA) and SigmaPlot (Jandel Scientific, USA). The amplitudes of eEPSC, non-NMDA- and NMDA-R currents before and during MPH application were compared statistically using a two-tailed paired Student’s t-test. Statistical significance was assessed at P<0.05. Asterisks in the figures indicate positive significance levels and “n” refers to the number of cells examined.

One way analysis of variance using SPSS software (IBM SPSS, USA) was performed on the behavioral pharmacological data. Post-doc Dunnett’s tests compared the drug application groups with saline control. Post doc LSD and Student-Neuman-Keuls multiple comparisons were evaluating the data between groups in the antagonism study.
